# Exploring the Larvicidal and Adulticidal Activity against *Aedes aegypti* of Essential Oil from *Bocageopsis multiflora*

**DOI:** 10.3390/molecules29102240

**Published:** 2024-05-10

**Authors:** Jefferson Rocha de Andrade Silva, Aimêe Almeida de Oliveira, Leandro Pereira França, Jefferson Diocesano da Cruz, Ana Claudia Fernandes Amaral

**Affiliations:** 1 Laboratório de Cromatografia, Departamento de Química, Instituto de Ciências Exatas, Universidade Federal do Amazonas, Manaus 69077-000, Brazil; aa.oliveira88@gmail.com (A.A.d.O.); francabio90@gmail.com (L.P.F.); 2 Laboratório de Plantas Medicinais e Derivados, Farmanguinhos, Fundação Oswaldo Cruz, Rio de Janeiro 21041-250, Brazil; jefferson_dacruz@hotmail.com

**Keywords:** adulticidal, *Aedes*, annonaceae, essential oil, larvicidal, molecular docking, sesquiterpenes

## Abstract

This study investigates the chemical composition of the essential oil obtained from the leaves of *Bocageopsis multiflora* (Mart.) R.E.Fr (Annonaceae), examining its effectiveness in combating both the larvae and adult forms of *Aedes aegypti* mosquitoes. Additionally, for a deeper understanding of the insecticidal activity, toxicity properties and molecular docking calculations were conducted using the main compounds of this essential oil. GC/MS analysis revealed the presence of 26 constituents, representing 95.2% of the essential oil, with the major components identified as the sesquiterpenes *α*-selinene, *β*-selinene, and *β*-elemene. Larvicidal assays demonstrated potent activity of this essential oil with significant LC_50_ values of 40.8 and 39.4 μg/mL at 24 and 48 h, respectively. Adulticidal assessments highlighted strong efficacy with LC_50_ of 12.5 µg/mL. Molecular docking analysis identified optimal interaction activities of *α*-selinene and *β*-selinene with key *Aedes* proteins. The in silico studies comparing synthetic insecticides with the major sesquiterpenes of the essential oil revealed that *β*-selinene exhibited a significantly higher binding affinity compared to the other two sesquiterpenes. Also, ADMET studies of the three main sesquiterpenes indicated acceptable drug-like properties. In these findings, safety evaluations showed low toxicity and skin sensitization for the main sesquiterpenes, contrasting with commercial synthetic insecticides. Therefore, in silico analyses suggest promising interactions with *Aedes* proteins, indicating its potential as an effective alternative to conventional insecticides These results show the larvicidal and adulticidal potential of the essential oil from *Bocageopsis multiflora* against *Aedes aegypti*, supported by its predominant constituents, *α*-selinene, *β*-selinene and *β*-elemene.

## 1. Introduction

Mosquitoes are a major global health concern, serving as the primary vector for a range of pathogens that cause diseases, particularly in tropical and subtropical regions. These diseases include malaria, filariasis, yellow fever, dengue, chikungunya, and Zika virus [1]. The World Health Organization (WHO) estimates that vector-borne diseases account for more than 17% of all infectious diseases, causing more than 700,000 deaths annually [2]. 

Given this context, arboviruses have become important and constant threats in tropical regions due to rapid climate changes, deforestation, population migration, disorderly urbanization, and precarious sanitary conditions that favor viral amplification and transmission [3,4,5]. The *Aedes aegypti* mosquitoes are efficient transmitters of dengue, chikungunya, and Zika diseases, which significantly impact public health [3]. Zika virus infection is known to lead to Guillain-Barré syndrome and negative pregnancy outcomes. These include a higher likelihood of preterm birth, fetal death, stillbirth, and a group of congenital malformations known as congenital Zika syndrome (CZS) in its most severe form. CZS encompasses microcephaly and other cranial abnormalities [4,5,6]. The number of cases of chikungunya and Zika have been reported in more than 89 countries across Asia, the Americas, and Africa [7,8]. Additionally, it is important to highlight that in the year 2022, 271,176 cases of chikungunya were reported, including 95 deaths in the Americas [9]. As for the epidemiology of dengue in Brazil, it is one of the main endemic diseases in the country. More than 5,867,255 cases were reported between 2014 and 2019, with the highest number of cases (1,696,340) occurring in 2015 [10]. There was a significant growth in the number of dengue fever cases in Brazil in 2019, which represents a public health problem [10]. It is important to mention that according to data from the World Health Organization (WHO), Brazil reported nearly 3 million cases in 2023, out of a global total exceeding 5 million cases [11].

Presently, the primary approach to controlling mosquito populations relies heavily on synthetic insecticides, including chlorinated hydrocarbon compounds, organophosphates, carbamates, pyrethroids, neonicotinoids, formamidines, and other molecules, plus botanical and microbial agents, and insect growth regulators like diflubenzuron and methoprene [12]. However, the continuous and extensive use of these chemicals has led to an increase in resistance among vector populations [13,14]. Furthermore, these compounds can cause adverse effects on non-target organisms and also present risks to human health [1]. In response to these challenges, alternative control methods have been proposed. These include the use of biopesticides, such as *Bacillus thuringiensis* and *B. sphaericus* [15,16,17] and, another strategy involves the creation of sterile insects through radiation, a technique that reduces the ability of these insects to reproduce [17,18]. Transgenic technologies, which involve the genetic modification of vectors, are also being explored as potential control methods [17,19].

In response to concerns about chemical resistance and environmental contamination associated with conventional insecticides, plant-derived alternatives such as essential oils (EOs) and their secondary metabolites are established and represent a reasonable framework of scientific research aimed at controlling vector insects [20,21]. EOs offer significant promise to improve insect vector control due to their inherent advantages: low toxicity, multi-target efficacy due to the presence of various bioactive compounds, and relatively low raw material costs [20,22]. 

In this context, essential oils obtained from plant species of the Annonaceae family exhibit various bioactivities, such as antimicrobial activity [23], cytotoxic activity [24], and activity against *Ae. aegypti* larvae [25]. Inspired by the biological activity demonstrated by essential oils (EOs) from species of this family, we turned our attention to the genus *Bocageopsis*. This genus encompasses four species: *B. mattogrossensis*, *B. canescens*, *B. multiflora*, and *B. pleiosperma*, all restricted to South America, with a presence in the Amazon region of Brazil, specifically in the states of Amazonas and Pará [26].

The chemical exploration of the *Bocageopsis* genus has involved the characterization and assessment of the essential oils extracted from its leaves. These EOs have been evaluated for their antibacterial properties [23,24] and cytotoxic effects [25]. The chemical composition of the essential oils revealed terpenes such as *β*-bisabolene, bergamotene, *β*-farnesene, *β*-selinene, *α*-selinene, and farnesol as their main constituents [27,28,29]. In articles specifically addressing *B. multiflora* (Mart.) R.E.Fr, it has been reported that the essential oils from this species demonstrate activity against the promastigote forms *of Leishmania amazonensis* [27], as well as exhibiting bactericidal and antimicrobial activity [23,29].

Following on from these findings, the application of molecular docking analysis in assessing the insecticidal or larvicidal potential of essential oils and their key constituents against proteins of *Aedes* sp. represents a cutting-edge approach to mosquito control [30]. Recent studies have employed molecular docking to uncover the complex interactions between constituents of essential oils and crucial mosquito proteins. Researchers have focused on proteins involved in the nervous system, reproductive processes, and metabolic pathways of insects [31]. A noteworthy example involves the use of molecular docking to assess the binding affinity of specific compounds found in essential oils to the acetylcholinesterase of *Aedes aegypti*, a vital enzyme in the mosquito’s nervous system [32]. It is also employed to analyze their effects on crucial targets within the mosquito life cycle, such as sterol transporter protein-2 (SCP-2), which is predominantly expressed in the midgut tissue of larvae and plays a crucial role in the absorption of cholesterol during feeding stages, thus being essential for insect metabolism [33,34,35]. In this context, examining the impacts of compounds found in essential oils through molecular docking on key targets within the mosquito life cycle helps in identifying promising compounds for further investigation.

In light of the outlined scenario, this study showcases the chromatographic profile of the essential oil obtained from *Bocageopsis multiflora*. Furthermore, it includes assessments for larvicidal and adulticidal effects against *Aedes aegypti*. Additionally, the study incorporates molecular docking analyses involving the three main compounds, *β*-elemene, *α*-selinene, and *β*-selinene identified in the essential oil of *B. multiflora* and three key proteins in the insect’s metabolism. In silico studies were also employed to evaluate ADMET properties, with the aim of elucidating the mode of action and predict the safety profile of these secondary metabolites. The results obtained in this work illustrate the significant potential of this essential oil and its constituents to play a vital role in developing future insecticides and in new strategies for mosquito control.

## 2. Results and Discussion

### 2.1. GC/MS Analysis of the Essential Oil of B. multiflora

The extraction of essential oil from *B. multiflora* represents an expanding area of studies fueled by the diverse chemical composition and biological properties observed in essential oils from species within the Annonaceae family. This interest stems from the remarkable antimicrobial, cytotoxic, and larvicidal actions demonstrated by essential oils of different members of this family against *Ae. aegypti* larvae [23,24,25,27,29]. 

In this study, the hydrodistillation of *B. multiflora* leaves resulted in essential oil with a slightly yellowish hue and a pleasant aroma, showing a yield of 1.3% *w*/*v*. Table 1 presents the chemical composition of this essential oil. Gas chromatography/mass spectrometry (GC/MS) analysis of the chromatogram for the EO revealed the presence of 26 constituents, with the main constituents being the sesquiterpenes *β*-elemene (28.8%), *α*-selinene (9.8%), and *β*-selinene (8.6%). The chromatographic profile of an essential oil derived from the same plant species can vary due to various factors, such as abiotic and biotic influences. The chemical composition of essential oils may undergo changes due to environmental factors such as soil type, climate, and altitude [36,37]. These factors can impact the plant’s metabolism, leading to variations in the synthesis of secondary metabolites, including essential oils [36].

In a comparative analysis between the essential oil obtained from *B. multiflora* leaves collected in Rondônia, Brazil, and the essential oil obtained in our study, significant quantitative and qualitative discrepancies were observed in the main constituents. Particularly, sesquiterpenes were found to be predominant in different essential oils derived from *B. multiflora* leaves. The essential oil obtained from *B. multiflora* leaves collected in the city of Porto Velho, State of Rondônia, Brazil, exhibited 1-epi-cubenol (16.6%) as the main sesquiterpene constituent [23]. On the other hand, a study conducted with the EOs from leaves collected in the state of Amazonas during two different climatic seasons showed variations in the sesquiterpene profile. The main constituent during the rainy season was bisabolene (13.2%), while during the dry season, it was spathulenol (16.2%) [27].

### 2.2. Larvicidal and Adulticidal Activity

The LC_50_ and LC_90_ values of *B. multiflora* EO against *Ae. aegypti* larvae after 24 and 48 h of application are listed in Table 2. 

Taking into account the mortality rate for each concentration, the median lethal concentration (LC_50_) obtained qualifies the essential oils as potential larvicidal agents. Then, the essential oils with an LC_50_ value below 100 μg/mL are deemed effective larvicidal agents, while those with an LC_50_ below 50 μg/mL are classified as highly active [38,39]. In this context, the essential oil from *B. multiflora* leaves demonstrated significant activity against *Ae. aegypti* larvae in the analyses at 24 and 48 h, with LC_50_ values of 40.8 μg/mL and 39.4 μg/mL, respectively. The observed activity could be attributed to the synergy among the constituents with higher content: *β*-selinene (8.6%), *α*-selinene (9.8%), and *β*-elemene (28.0%). According to literature data, *β*-selinene demonstrates an LC_50_ value of 136.0 μg/mL against *Ae. aegypti* larvae [40], which is higher than our findings. Moreover, *β*-elemene exhibits notably superior activity, evidenced by a LC_50_ of 11.15 μg/mL against *Ae. albopictus* larvae after 24 h exposure [40].

Many studies in the literature are devoted to investigating the effectiveness of essential oils in the ongoing search for new larvicides. For instance, recently, Luz and coworkers [41] conducted a review encompassing 337 essential oils from 225 plant species with bioactivity against *Ae. aegypti* larvae. For a better understanding of the connection between essential oils and their larvicidal effects, some studies examine the effectiveness of the main compounds in these oils and show out that the whole essential oil is more active than its main compounds [42]. These reports hold significance and can be correlated with the findings regarding the efficacy of *B. multiflora* essential oil as a larvicide against *Ae. aegypti*.

Another interesting approach of the present work was the results regarding the biological activity against adult females of *Ae. aegypti* that are described in Table 3. 

The EO obtained from *B. multiflora* leaves exhibited excellent activity against adult *Ae. aegypti*, with an LC_50_ of 12.5 µg/mL. This result is noteworthy as it falls below the threshold indicated in the literature for highly active essential oils, i.e., those with LC_50_ < 50 µg/mL [38,39,43]. This outcome is quite promising and aligns with the ongoing search for plant-based insecticides. Indeed, a number of studies show interest in researching natural products as alternative strategies against insects that are important for public health [22,44,45]. The primary motivation for the interest in natural products, specifically essential oils, lies in their considerable advantages compared to synthetic insecticides. These include low toxicity to mammals, minimal environmental impact, diverse modes of action, and a low likelihood of inducing vector resistance due to the inherent complexity of essential oil composition [22,44]. However, despite their promising activities, establishing a complex mixture of essential oils as a viable insecticide is challenging, making it unlikely, from a commercial standpoint, that any single EO will be utilized for insect control without further research. Several factors must be considered, such as the availability of raw materials, the preparation and formulation of bioinsecticides with controlled chemical compositions, and the identification of active constituents before labeling an EO as a potential agent against mosquitoes [22,45]. Isman [22] recently discussed the development of a bioinsecticide meeting commercial demands, emphasizing the importance of defining procedures for formulating insecticidal products. Nanotechnology holds promise for enhancing the stability and bioavailability of essential oils in these formulations, ensuring the persistence of volatile compounds and facilitating delivery through insect skin. Despite challenges in biomass availability, particularly regarding plant species cultivation, emerging alternatives are being explored to address this issue. However, the selection of an EO or its bioactive components requires further investigation for resolution, given the inherent challenges associated with each option.

In this context, the scarcity of literature data regarding the biological activity of *B. multiflora* against adult *Ae. aegypti* underscores the importance of investigating the properties and effectiveness of this plant essential oil for vector control purposes. The results of our study demonstrate that the essential oil derived from this plant is effective in eliminating *Ae. aegypti* in the larval and adult stages. After obtaining interesting results from experimental analyses on the larvicidal and adulticidal activities of *B. multiflora* essential oil, a molecular docking analysis was conducted between key enzymes of *Aedes* species and the three main compounds of the essential oil.

### 2.3. Molecular Docking

Table 4 displays the findings from the molecular docking analysis of the three key *Aedes* species related proteins and the three main compounds, *β*-elemene, *α*-selinene, and *β*-selinene, present in the essential oil (EO) obtained from *B. multiflora* leaves. 

**Figure 1:**
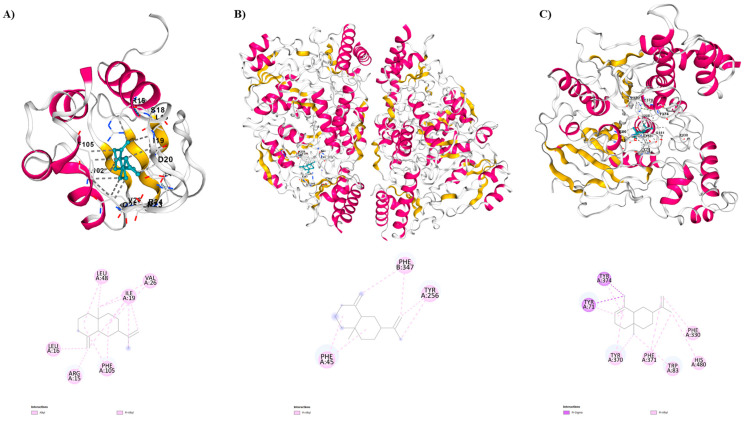
Two-dimensional and three-dimensional intermolecular contact between EOs compounds: (**A**) *β*-selinene against Sterol Carrier Protein-2 (PDB = 1PZ4). (**B**) *β*-selinene against *Aedes aegypti* kynurenine aminotransferase (PDB = 6MFB). (**C**) *α*-selinene against Acetylcholinesterase (PDB = 6XYU).

Regarding the insecticidal capability of the compounds identified in the EO, the isomers *α*-selinene and *β*-selinene exhibited excellent interaction activities with the three molecular targets of mosquitoes. In other words, the likelihood of action on vital mosquito proteins was more comprehensive, as these sesquiterpenes demonstrated strong interactions with all three receptors. This indicates that these compounds may be responsible for the observed activity. In all three scenarios, these two sesquiterpenes are the ones that interact most with the amino acid residues of the respective proteins, with a predominance of hydrophobic interactions for the three target proteins (Figure 1). This suggests that incorporating these ligands as constituents of a bioinsecticide is likely to impact the vector through diverse mechanisms, leading to insecticidal effects.

When compared to insecticides such as temephos (−7.8 kcal/mol) and deltamethrin (−8.9 kcal/mol), as well as the other main compounds of the *B. multiflora* essential oil, *β*-selinene exhibited a greater binding affinity value (−9.1 kcal/mol) with the target protein Sterol Carrier Protein-2 (PDB = 1PZ4). In this context, the comparison among the sesquiterpenes shows that *β*-selinene exhibited interactions between the *α*-helix residues LEU102-PHE105, GLU103 and ARG15, as well as between the *β*-sheet residues LEU16-SER18, LEU48, ILE19-ASP20, and GLN25-VAL26. The observed interactions are van der Waals, alkyl, and π-alkyl. Additionally, the sites of the interactions observed with the amino acid residues of AeSCP-2 resemble the sites described for the sterol carrier protein inhibitor-1 (SCPI-1; Arg15, Gln25, Val26, Phe32, Leu64, Leu102, Gln103, Phe105) [46] thus providing partial justification for the high affinity observed for this sesquiterpene. 

A significant part of the insecticidal capacity of the main compounds presents in the EO obtained from *B. multiflora* leaves is derived from the mechanism of action on essential molecular targets in the insect life cycle. These ligands can competitively inhibit the cholesterol binding site of SCP-2, occupying the same protein binding region as cholesterol and preventing this binding, blocking the cholesterol metabolic pathway in the insect organism [47].

*β*-selinene demonstrated a significant binding affinity with a value of −8.0 kcal/mol, surpassing all the main sesquiterpenes found in the EO, as well as when compared to the positive control temephos (−7.4 kcal/mol) in molecular docking studies with 3-hydroxykynurenine transaminase (PDB = 6MFB). Also, *β*-selinene showed interactions between the *α*-helix residues PHE45, HIS46, ASP47, in chain A, while in chain B interactions were observed between the *α*-helix residues ILE103, TRP328, TRP329, SER332, MET336, PHE347, GLY348, and MET351and between the *β*-sheet residues GLU342, GLN344, GLY345, ARG356. The observed interactions are π-alkyl.

The interactions between the ligand, *β*-selinene, and the HKT active sites, particularly involving the amino acid residues SER43, ASN44, and PHE45, hold significant importance. Notably, these sites exhibit close proximity to the active residue ARG356, and molecular docking studies have revealed an interaction with the ligand. This finding enables us to delve into a plausible biological mechanism, suggesting the inhibition of HKT enzyme activity. This result is supported by previous research conducted by Rossi and collaborators (2006) as well as by Chen and collaborators (2022) [48,49].

Nature consistently surprises us. The metabolic processes involving tryptophan necessitate mosquitoes to employ biochemical tools, enabling them to host microorganisms harmful to human health, such as *Plasmodium falciparum*, the causative agent of malaria. In this context, the tryptophan oxidation process produces metabolites that, when accumulated, inflict damage on the insect’s organism [50]. In response to this, the mosquito employs the enzyme 3-hydroxykynurenine transaminase (HKT) to convert 3-hydroxykynurenine (3-HK) into xanthurenic acid [51]. This process not only bolsters the mosquito’s defense, averting adverse effects such as paralysis or death caused by 3-HK [50], but also serves as an antioxidant during the digestion of a blood meal [52]. Another remarkable aspect of this metabolic coordination is the importance of 3-HK in the pigmentation of the mosquito’s eyes. The organism needs to control the quantity of this compound to avoid self-intoxication, underscoring the complexities involved in maintaining a delicate balance [48]. Furthermore, it has been noted that the gut microbiota within *Anopheles stephensi* contributes to heightened resistance against *Plasmodium* infection. This resistance is facilitated by the synthesis of the enzyme kynureninase, which leads to the production of 3-HK [48,53]. Considering these findings, the HKT enzyme emerges as a substantial target in the quest for new insecticidal agents against *Aedes* species.

Acetylcholinesterase (AChE) is a pivotal enzyme in the metabolism of various organisms, including mosquitoes. In insects, AChE’s primary function is to hydrolyze the neurotransmitter acetylcholine, leading to the cessation of neuronal excitation in the postsynaptic membrane, resulting in paralysis and death of the invertebrate [45]. Additionally, other researchers suggest that the neurotoxic mode of action, impacting ion transport and AChE, as well as the disruption of octopamine, a crucial neurotransmitter, neurohormone, and neuromodulator in invertebrate systems, also contribute to the observed effects [54]. Scientific literature reports several studies highlighting the biological activity of natural products, such as essential oils, on AChE [55]. Consequently, these findings may suggest potential pathways for the action of essential oil of *B. multiflora* on AChE. 

In this research, the notable binding affinity exhibited by *α*-selinene (−9.7 kcal/mol) with the protein, approaching that of the deltamethrin (−10.9 kcal/mol), positions this sesquiterpene as a promising candidate for inclusion in a bioinsecticide targeting AChE. The *α*-selinene showed interactions between the *α*-helix residues TYR370-PHE371, and TYR374, and between the *β*-sheet residues TRP83, HIS480, TYR71, and PHE330. The interactions observed are Van der Waals, π-sigma, and π-alkyl. 

The binding with the amino acid residues of AChE (PDB = 6XYU) involve a considerable number of active sites for this enzyme, as elucidated by Harel et al. (2000) [56]. This provides a basis for considering an alternative mechanism of action due to the compounds present in the essential oils (EOs) of *B. multiflora*. 

### 2.4. Prediction of the ADMET Properties of the Main Substances of the EO of B. multiflora

The three main compounds of *B. multiflora* essential oil were also evaluated through in silico study to predict ADMET properties as listed in Table 5. In this context, all sesquiterpenes were deemed acceptable according to Lipinski’s rules, which establish that a drug-like compound must have a molecular weight (MW) ≤ 500 Da, a logarithm of the n-octanol/water partition coefficient (logP) ≤ 5, a number of hydrogen bond donors (HBD) ≤ 5, and a number of hydrogen bond acceptors (HBA) ≤ 10 [57].

The plasma protein binding (PPB) of *α*-selinene exceeded 90%, albeit lower than that of the larvicide temephos and adulticide deltamethrin. This indicates that this sesquiterpene might possess a low therapeutic index as the duration of stay in the body might be low [58]. 

Concerning metabolism, most of the compounds evaluated inhibit the CYP2C19 and CYP2C9 isoenzymes. These two cytochrome P450 enzymes (CYPs) are estimated to be responsible for roughly 35–40% of drug oxidative metabolism and approximately 25% of overall xenobiotic biotransformation. Xenobiotics encompass a broad range of compounds derived from diverse sources, including environmental contaminants and industrial pollutants [59]. 

The evaluation of various toxicity parameters, such as AMES toxicity, carcinogenicity, reproductive effects, and hepatotoxicity, confirmed the safety of the main sesquiterpenes found in *B. multiflora* essential oil. When considering their potential use in products applied to the skin, such as repellent lotions, or bioinsecticides, it is crucial to assess skin sensitization and eye irritation. In this context, all sesquiterpenes assessed through in silico analysis showed skin sensitization values below 0.8, indicating that they are non-sensitizers. Conversely, the insecticides temephos (for larvae) and deltamethrin (for adults) exhibited values of 0.936 and 0.874, respectively, classifying them as sensitizers. 

Synthetic insecticides not only have undesired effects on non-target organisms but also lead to the selection of resistant vector insects and pests [60,61], like *Ae. aegypti* and *Ae. albopictus* [62,63]. Moreover, these synthetic commercial products can be harmful to human health and the environment (contamination of water, air, and soil resources) due to intensive and prolonged application [64]. Therefore, it is crucial to find environmentally safe alternatives that are potentially more effective and suitable for use in *Aedes* control programs. 

In our in silico study, the aquatic toxicity of the three main sesquiterpenes of EO were characterized by fathead minnow LC_50_ (96 h), *Tetrahymena pyriformis* IGC_50_ (48 h), and *Daphnia magna* EC_50_ (48 h). The LC_50_, IGC_50_, and LC_50_DM of *α*-selinene, *β*-selinene, and *β*-elemene are 4.1, 4.7, and 3.6 (μg/mL), 5.6, 6.7, and 5.4 (μg/mL), and 6.2, 6.0, and 6.6 (μg/mL), respectively. In general, when compared to results from other studies, all the sesquiterpenes exhibited low ecotoxicological profiles against the selected organisms [65].

## 3. Materials and Methods

### 3.1. Reagent and Chemicals

Temephos and dimethyl sulfoxide (DMSO) were obtained from Sigma-Aldrich Corporation (St. Louis, MO, USA). n-Alkane standard (C7–C30) was purchased from Supelco (Bellefonte, PA, USA). Distilled water was used for general procedures. Solvents (dichloromethane, dimethyl sulfoxide (DMSO), ethanol, and methanol) were of analytical and chromatographic grades (Columbus, OH, USA, TEDIA).

### 3.2. Plant Material and Extraction of Essential Oil 

The leaves of *Bocageopsis multiflora* were gathered in May 2018 from the Adolpho Ducke Forest Reserve, Km 26 Manaus, Itacoatiara highway, in the State of Amazonas, Brazil. These leaves, weighing 200 g, were chopped and subjected to hydrodistillation for 3 h using a modified Clevenger-type apparatus. The obtained EO was transferred into amber glass flasks and stored in the freezer for future use. Yield was determined based on the weight of the leaves utilized.

### 3.3. Rearing of Mosquitoes 

The insects, *Aedes aegypti*, were obtained from colonies established in the Laboratory of Malaria and Dengue of the National Institute for Amazonian Research (INPA, Manaus, Brazil). The colonies were maintained without exposure to insecticides, under controlled conditions at room temperature (26 ± 2 °C) and a relative humidity of 70–85%, with a photoperiod of 12:12 h (light/dark). The procedure was performed using the methodology described by Thanigaivel et al. [66]. The eggs of *A. aegypti* obtained from spawning colonies were placed in containers with water provided for the larvae to hatch. The larvae were raised in a plastic tray that contained distilled water and were fed with a mixture of cat food (Whiskas^®^) and bovine liver powder in a 1:1 ratio. The larvae were kept until they reached the third larval instar, and subsequently, a number were selected for the larvicidal bioassays. The remaining larvae were left in enameled basins until they reached pupation. The pupae were transferred to round plastic containers (50 mL), containing distilled water, and were placed in breeding cages (dimensions 30 cm × 30 cm × 30 cm) for the emergence of adults. The adults were fed with a 10% sucrose solution and the blood meal, according to the CEUA protocol 054/2018 of the INPA Ethics Committee on Animal Use. Blood-fed females, 2 to 5 days old, were used in the adulticidal bioassays.

### 3.4. Larvicidal Bioassay

Eggs and adults of *Ae. aegypti* of Rockefeller strain were acquired at the Insectarium of the Laboratory of Malaria and Dengue of the National Institute for Research in the Amazon (INPA). The eggs were immersed in deionized water for hatching. Fish feed (Whiskas^®^) was provided ad libitum and the larval water was replaced twice a week. After hatching, the larvae were monitored for 3 to 4 days until they reached the third and fourth stages of development to be used in the larvicidal assays. The mosquito colonies were maintained at 28 ± 2 °C and relative humidity of 80 ± 10% and provided with 10% sucrose solution. According to CEUA Protocol 054/2018 of the Ethics Committee for the Use of Animals, INPA, the animals were anesthetized for 10 min to feed the insects. The females were fed once a week with blood from a hamster to promote egg development. These eggs were collected on wet filter paper surfaces once a week and stored for incubation. The larvicidal assays were performed according to the susceptibility test [67]. Ten larvae at the end of the third to the beginning of the fourth instar were used for the tests. The EO was tested at five different concentrations (100, 75, 50, 25, and 10 μg/mL) diluted in 1 mL of a solution containing 1.0% (*v*/*v*) dimethyl sulfoxide (DMSO). All experiments were carried out in quintuplicate with positive control (Temephos^®^ at 0.12 μg/mL), negative control (1.0% (*v*/*v*) DMSO in distilled water), and the five different concentrations. After 24 and 48 h, live and dead larvae (immobile individuals and/or those deposited on the bottom of the glass) were counted. Mortality data were corrected, if necessary, using Abbott’s (1925) formula [68]. 

### 3.5. Adulticidal Bioassay 

The adulticidal bioassay was carried out according to the guidelines of Brogdon and Chan [69]. The essential oil was solubilized in acetone to obtain five concentrations (50, 20, 15, 10, and 7 μg/mL). Glass bottles of 295 mL capacity were used for the test and 1.0 mL of each concentration was added to the flask. Acetone and deltamethrin (0.60 μg/mL) were used as negative and positive controls, respectively. The contents of each bottle were gently agitated by turning to coat all sides of the bottle. Then the caps were removed and the bottles were continuously rolled on their sides until all the liquid disappeared. The bottles were left horizontally for 12 h. Using a mouth aspirator, 15 female mosquitoes were introduced into each test bottle, and the control. The number of alive and dead mosquitoes was registered after 90 min., with readings every 15 min. In the test, a mosquito is classified as alive if it is able to fly regardless of the number of legs still intact; and it is considered dead or knocked over if it is immobile, unable to fly, or stand in a coordinated manner. All bioassays were performed at 28 ± 2 °C and relative humidity of 80 ± 10%. The experiments were performed in three replications, together with the control. Mortality data were corrected, if necessary, using Abbott’s (1925) formula [68]. 

### 3.6. Essential Oil Analysis Using Gas Chromatography/Mass Spectrometry (GC/MS)

The identification of the constituents of the essential oil was performed by comparison of their retention indices and mass spectra against those reported in the literature [70] or those presented in the Wiley library (version 7.0) for the GC/MS equipment. The retention indices were calculated for all volatile constituents using n-alkane homologous series. GC/MS analyses were performed using a Shimadzu gas chromatograph interfaced with a HP 5973N Mass Selective Detector (ionization voltage 70 eV), equipped with a DB-5MS capillary column (30 m × 0.25 mm, film thickness 0.25 μm), using helium as carrier gas (1.0 mL min^−1^). The oven temperature was programmed from 50 to 290 °C at a rate of 3 °C min^−1^, then isothermal heating at 290 °C for 10 min, using He as the carrier gas (1.0 mL min^−1^). Injector and detector temperatures were 230 °C and 290 °C, respectively. Injection volume was 1.0 μL (2 mg of the sample in 1 mL of CH_2_Cl_2_), in splitless mode. Linear velocity (ū) was 14 cm s^−1^. MS interface temperature was 280 °C; mass range was 40–700 μ; scan speed was 150 μs^−1^; interval was 0.50 s (2 Hz).

### 3.7. Acquiring Protein Structures 

The AeSCP-2 protein (PDB = 1PZ4), 3-hydroxykynurenine transaminase (HKT) (PDB = 6MFB), and the crystallographic structure of *Drosophila melanogaster* acetylcholinesterase (AChE) in complex with the tacrine derivative, 9-(3-iodobenzylamino)-1,2,3,4-tetrahydroacridine (PDB = 6XYU), were obtained from the database https://www.rcsb.org/, accessed on 15 January 2024. All structures were downloaded in PDB format. 

### 3.8. Selection and Construction of 3D Ligand Structures

The three main compounds identified in the essential oil obtained from *Bocageopsis multiflora* were selected for molecular docking: *α*-selinene ((3*R*,4a*R*,8a*R*)-5,8a-dimethyl-3-prop-1-en-2-yl-2,3,4,4a,7,8-hexahydro-1*H*-naphthalene); *β*-selinene ((3*R*,4a*S*,8a*R*)-8a-methyl-5-methylidene-3-prop-1-en-2-yl-1,2,3,4,4a,6,7,8 octahydronaphthalene); *β*-Elemene ((1*S*,2*S*,4*R*)-1-ethenyl-1-methyl-2,4-bis(prop-1-en-2-yl)cyclohexane). Temephos ([4-(4-dimethoxyphosphinothioyloxyphenyl)sulfanylphenoxy]-dimethoxy-sulfanylidene-λ5-phosphane), and deltamethrin ((*S*)-cyano (3-phenoxyphenyl)methyl (1*R*,3*R*)-3-(2,2-dibromoethen-1-yl)-2,2-dimethylcyclopropane-1-carboxylate) were used for comparison purposes in the docking score.

The acquisition of isomeric smiles of the ligands was performed from the database: https://pubchem.ncbi.nlm.nih.gov/, accessed on 15 January 2024. Subsequently, the structures were drawn using the ChemDraw Professional 12.0 molecules software, and through the software extension, Chem3D 12.0, the conversion and visualization of the structures in 3D were performed. With the obtained three-dimensional structures of the ligands, calculations were carried out in MM2 for energy minimization. Once the 3D molecules with minimized energy were obtained, they were saved in mol2 format in a single folder for docking analysis.

### 3.9. Molecular Docking

Molecular docking simulations were conducted through blind docking procedures using the online server CB-Dock2 [71,72]. CB-Dock is a ligand-protein blind docking server that employs a cavity detection approach based on protein surface curvature to guide molecular docking with AutoDock Vina. The enhanced version, utilized in this study, CB-Dock2, integrates cavity detection, docking, and homology model fitting to increase accuracy in identifying the binding site and predicting the binding pose.

#### Analysis of Intermolecular Interactions and Figure Construction

The results of molecular docking, i.e., the generated poses of interactions between receptor and ligand, were assessed using the Discovery Studio Visualizer v21.1.0.20298 molecular visualization software.

### 3.10. Prediction of Properties of ADMET

ADMET filtering analysis of all ligands selected for docking was predicted using the ADMETlab 2.0 web server [https://admetmesh.scbdd.com/service/evaluation/index (accessed on 15 January 2024)] and a SwissADME^®^ [http://www.swissadme.ch (accessed on 16 January 2024)]. The isomeric SMILES of the ligands downloaded from the PubChem database (https://pubchem.ncbi.nlm.nih.gov/, accessed on 15 January 2024) were used to calculate the ADME/tox parameters in standard mode.

### 3.11. Statistical Analysis

The lethal concentration that kills 50% of larvae or adults (LC_50_) and the lethal concentration that kills 90% of larvae or adults (LC_90_) were determined by probit regression with 95% fiducial limits using the POLO PLUS^®^ program (LeOra Software version 1.0, Berkeley, CA, USA) [73,74]. Significant differences were determined by analysis of variance (bidirectional ANOVA) followed by Tukey’s test (*p* < 0.01 and *p* < 0.05) using BioEstat 5.0 Windows software (Belém, PA, Brazil). 

## 4. Conclusions

The study of the essential oil (EO) obtained from the leaves of *Bocageopsis multiflora* indicates the predominance of sesquiterpenes, which could be associated with the biological activities observed against the larvae and adults of *Aedes aegypti*. Molecular docking analysis revealed that the three sesquiterpenes, *α*-selinene, *β*-selinene and *β*-elemene. Specifically, *α*-selinene and *β*-selinene, exhibited optimal interaction activities with mosquito proteins, suggesting their potential role in insecticidal action. Notably, *β*-selinene showed higher binding affinity than conventional insecticides, indicating its promising use in bioinsecticide formulations. According to docking and ADMET results, the three main sesquiterpenes appear to be safe for use. This study emphasizes their safety compared to commercial insecticides, stressing the importance of environmentally friendly alternatives for effective *Aedes* control. Overall, the essential oil from *B. multiflora* shows promise as an eco-friendly option for managing *Aedes aegypti* populations, supported by its chemical composition, insecticidal effects, favorable ADMET properties, and minimal ecotoxicological impact. However, further research is needed to assess its practical integration into mosquito vector control programs.

## Figures and Tables

**Table 1:** Chemical composition of the essential oils obtained from leaves of *Bocageopsis multiflora*.

Components ^a^	RI ^b^	RI ^c^	Essential Oil (%)
Sesquiterpenes			
bicycloelemene	1327	1323	6.21
isoledene	1374	1371	0.16
cis-*β*-elemene	1382	1381	2.07
*β*-elemene	1389	1392	28.83
*α*-gurjunene	1409	1407	0.55
*α*-bergamotene	1411	1410	3.54
trans-caryophyllene	1417	1418	1.46
γ-elemene	1434	1433	4.83
aromadendrene	1439	1437	1.21
*α*-humulene	1452	1449	1.51
γ-gurjunene	1475	1474	0.33
*β*-chamigrene	1476	1477	3.78
germacrene D	1480	1481	1.18
*β*-selinene	1489	1486	8.64
ledene	1496	1495	0.71
*α*-selinene	1498	1499	9.84
*β*-bisabolene	1505	1505	0.36
δ-guaiene	1509	1508	2.51
*β*-sesquiphellandrene	1521	1520	0.29
δ-cadinene	1522	1522	0.65
globulol	1590	1589	2.51
viridiflorol	1592	1591	3.49
ledol	1602	1603	1.52
γ-eudesmol	1630	1631	0.76
neointermedeol	1658	1655	7.94
eudesm-7(11)-en-4-ol	1700	1701	0.29
Total			95.17

^a^ Components are listed according to their elution from a DB-5MS column; ^b^ Literature RI taken from NIST 16 or ADAMS libraries; ^c^ Retention index based on a homologous series of normal alkanes.

**Table 2:** Larvicidal activity of *Bocageopsis multiflora* essential oil against *Aedes aegypti*.

Plant	24 h						48 h					
*B. multiflora*	LC_50_ (µg/mL)	CI 95% (LCL–UCL)	LC_90_ (µg/mL)	CI 95% (LCL–UCL)	Slope	*X* ^2^	LC_50_ (µg/mL)	CI 95% (LCL–UCL)	LC_90_ (µg/mL)	CI 95% (LCL–UCL)	Slope	*X* ^2^
	40.8	(35.1–51.3)	71.5	(55.4–127.0)	5.260	150.81	39.4	(34.3–47.8)	68.7	(54.4–111.6)	5.307	133.20

LC_50_ lethal concentration for 50% of larvae, LC_90_ lethal concentration for 90% of larvae, 95% CI confidence interval of 95%, LCL lower confidence limit, UCL upper confidence limit. Chi-squared value (*X*^2^).

**Table 4:** Molecular docking scores of five major compounds of essential oil of *Bocageopsis multiflora* against mosquito proteins (PDB = 1PZ4; 6MFB; 6XYU).

Sterol Carrier Protein-2–AeSCP-2 (PDB = 1PZ4)		
Compounds	Binding Affinity (kcal/mol)	Amino Acid Residues
*α*-selinene	−8.0	ARG15 LEU16 ILE19 VAL26 MET46 LEU48 LEU101 LEU 102 PHE105 ILE106 SER108
*β*-selinene	−9.1	ARG15 LEU16 SER18 ILE19 ASP20 GLN25 VAL26 LEU48 LEU102 GLU103 PHE105
*β*-elemene	−6.5	ARG15 LEU16 ILE19 ARG24 GLN25 VAL26 MET46 LEU48 LEU 102 PHE105 SER108
Temephos	−7.8	ARG15 LEU16 SER18 ILE19 ASP20 ASN23 GLN25 VAL26 LEU48 LEU 102 PHE105
Deltamethrin	−8.9	ARG15 LEU16 SER18 ILE19 ASP20 GLN25 VAL26 LEU48 LEU102 PHE105 LEU109
3-hydroxykynurenine transaminase (PDB = 6MFB)		
*α*-selinene	−7.9	Chain A: SER43 ASN44 PHE45 HIS46 ASP47 Chain B: TRP328 TRP329 SER332 GLU342 GLN344 GLY345 PHE347 MET351 ARG356
*β*-selinene	−8.0	Chain A: SER43 ASN44 PHE45 HIS46 ASP47 Chain B: ILE103 TRP328 TRP329 SER332 MET336 GLU342 GLN344 GLY345 PHE347 GLY348 MET351 ARG356
*β*-elemene	−5.9	Chain C: TRP329 SER332 GLU342 GLN344 GLY345 GLY346 PHE347 MET351 ARG356 Chain D: SER43 PHE45 HIS46 PHE50 GLN253
Temephos	−7.4	Chain A: SER43 ASN44 PHE45 HIS46 ASP47 PHE50 GLN253 LYS254 ARG255 TYR256 Chain B: ILE103 TRP104 LYS205 TRP328 TRP329 SER332 MET336 GLU342 GLN344 GLY345 PHE347 GLY348
Deltamethrin	−10	Chain C: ILE103 TRP104 ARG107 TRP328 TRP329 SER332 MET336 GLU342 ILE343 Chain D: SER43 ASN44 PHE45 HIS46 ASP47 GLU48 PHE50 GLN253 LYS254 ARG255
Acetylcholinesterase (PDB = 6XYU)		
*α*-selinene	−9.7	TYR71 TRP83 GLY149 GLY150 GLY151 THR154 SER238 PHE330 TYR370 PHE371 TYR374 HIS480
*β*-selinene	−8.2	TYR71 GLY79 GLU80 TRP83 GLY149 GLY150 THR154 GLY155 TYR370 HIS480
*β*-elemene	−8.0	TYR71 GLY79 GLU80 TRP83 ASN84 GLY149 TYR370 TYR374
Temephos	−6.0	THR275 LYS278 LEU328 SER329 PHE330 ALA333 LYS403 GLU408 HIS438 PHE439
Deltamethrin	−10.9	GLY155 LEU159 TYR162 GLU237 SER238 TYR324 PHE330 TYR370 PHE371 TYR374

**Table 5:** In-silico ADMET profile of the selected ligands.

Properties	Compounds				
	*α*-Selinene	*β*-Selinene	*β*-Elemene	Temephos	Deltamethrin
Lipinski rule	Accepted	Accepted	Accepted	Accepted	Accepted
Absorption					
PPB (%)	95.7	89.7	85.4	103.4	97.7
BBB permeation	No	No	No	No	Yes
GI absorption	Low	Low	Low	Low	High
Log *K*_p_ (skin permeation; cm/s)	−3.85	−3.68	−3.21	−4.91	−4.98
Physicochemical Properties					
Molecular Weight (g/mol)	204.35	204.35	204.35	466.47	505.20
Num. H-bond acceptors	0	0	0	6	4
Num. H-bond donors	0	0	0	0	0
logP	5.19	4.757	4.998	5.734	6.337
Metabolism					
CYP3A4 inhibitor	No	No	No	No	Yes
CYP2D6 inhibitor	No	No	No	No	No
CYP1A2 inhibitor	No	No	No	Yes	Yes
CYP2C19 inhibitor	Yes	Yes	Yes	Yes	Yes
CYP2C9 inhibitor	Yes	Yes	Yes	Yes	Yes
Toxicity					
AMES Toxicity	Negative	Negative	Negative	Medium risk	Negative
Eye Irritation	Irritant	Irritant	Irritant	Irritant	Irritant
Carcinogencity	Non-carcinogens	non-carcinogens	non-carcinogens	non-carcinogens	non-carcinogens
Skin Sensitization	0.583	0.274	0.225	0.936	0.874
Reproductive effects	Low risk.	Low risk.	Low risk.	High risk.	-
Hepatotoxicity	Non toxic	Non toxic	Non toxic	Non toxic	toxic

**Table 3:** Adulticidal activity of essential oils against *Aedes aegypti* females after 90 min of exposure.

Plant Species	LC_50_ (µg/mL)	CI 95% (LCL–UCL)	LC_90_ (µg/mL)	CI 95% (LCL–UCL)	Slope	*X* ^2^
*Bocageopsis multiflora*	12.5	(11.6–13.3)	17.1	(15.7–19.3)	9.258	5.911

LC_50_ lethal concentration for 50% of larvae, LC_90_ lethal concentration for 90% of larvae. 95% CI confidence interval of 95%, LCL lower confidence limit, UCL upper confidence limit. Chi-squared value (*X*^2^).

## Data Availability

Data are contained within the article.
